# Nanobiomaterials Used in Cancer Therapy: An Up-To-Date Overview

**DOI:** 10.3390/molecules24193547

**Published:** 2019-09-30

**Authors:** Iulia Ioana Lungu, Alexandru Mihai Grumezescu, Adrian Volceanov, Ecaterina Andronescu

**Affiliations:** 1 Faculty of Applied Chemistry and Materials Science, University Politehnica of Bucharest, 011061 Bucharest, Romania; iulia.lunguu@gmail.com (I.I.L.); avolceanov@yahoo.co.uk (A.V.); ecaterina.andronescu@upb.ro (E.A.); 2 National Institute of Laser, Plasma and Radiation Physics (NILPRP), Bucharest-Magurele, 077125 Magurele, Romania

**Keywords:** cancer therapy, biological barriers, nanoparticles, drug delivery, theranostics

## Abstract

The disadvantages that come with traditional cancer treatments, such as chemotherapy and radiotherapy, generated a research shift toward nanotechnology. However, even with the important advancements regarding cancer therapy, there are still serious stepping stones that need to be addressed. The use of both nanotechnology and nanomedicine has generated significant improvements in nano-sized materials development and their use as therapeutic, diagnosis, and imaging agents. The biological barriers that come from the healthy body, as well from the tumorous sites, are important parameters that need to be taken into consideration when designing drug delivery systems. There are several aspects of extreme importance such as the tumor microenvironment and vasculature, the reticuloendothelial system, the blood–brain barrier, the blood–tumor barrier, and the renal system. In order to achieve an effective system for cancer therapy, several characteristics of the nanoparticles have been outlined. Moreover, this review has also focused on the different types of nanoparticles that have been studied over the years as potential candidates for cancer therapy.

## 1. Introduction

Cancer is the primordial disease that causes a high rate of mortality worldwide. Classical cancer therapies that are still clinically used include chemotherapy, radiotherapy, and surgery. However, using chemotherapy or radiotherapy on their own has proven to have significant limitations. The main disadvantage of chemotherapy is its lack of specificity—apart from affecting cancerous cells, it also damages the surrounding cells/tissue—that leads to the development of multidrug resistance during the treatment, and the limitations can go as far as recurrence [1,2,3].

Even though there have been impressive improvements in the nanotechnology and biomedical field, cancer remains the most challenging disease to treat, still being the leading cause of mortality. There has been continuous growth in the number of deaths caused by cancer over the last years. Other diseases have also increased in mortality rates, such as heart or brain diseases; however, the increase is lower as compared to cancer [2].

Radiotherapy proved to be efficient when dealing with various cancers that are localized in specific body sites. However, this treatment also comes with several disadvantages varying from recurrence to morbidity. As mentioned above, the current treatments for cancer suffer from a lack of specificity that translates in the delivery of the anti-cancer agents in the targeted site at inadequate concentrations, high toxicity to the healthy and neighboring tissues and cells, and also inefficiency in tracking and managing reaction and side effects [4].

Nanotechnology is a union of several fields of science. It not only includes medicine, but also areas such as physics, chemistry, and molecular biology [5]. The main focus of nanotechnology is to investigate paths that have not been exploited yet regarding physical features, such as for instance magnetic, optical behaviors. Nanotechnology has the potential to overcome the disadvantages of conventional drug delivery by adjusting pharmacokinetics and delivery, resulting in diminishing side effects and therefore improving efficiency. When discussing the properties of nanoparticles in regard to drug delivery, surface area is an important factor to take into consideration. With an increased surface area, the quantity of anti-cancer agent that can be attached increases. This aspect and several other properties that will be discussed make them potential efficient candidates as drug delivery vectors [4].

With the development of nanotechnology, creating complex nanosystems that consist of two or more types of nanoparticles broadens the functionalization potential for drug delivery and imaging applications. Nanoparticles can vary in size ranging from 10 up to 400 nm, and can either encapsulate or attach to their surface various pharmacologically active drugs, depending on the surface properties [4].

There are several aspects that nanotechnology is researching for cancer treatment purposes, as following [6]:

- Increasing the efficiency of drug delivery and reducing side effects, therefore toxicity;
- Specific targeting of the active components in cell/tissues;
- Improving the properties of pharmacologically active drugs such as stability, solubility, half-life, and tumor aggregation;
- Generating stimuli-responsive drug release;
- Expanding the area of drugs encapsulated/attached to biomacromolecules such as proteins, mRNA;
- Improvement of therapeutic efficiency by delivering multiple active agents to a specific targeted site in order to overcome limitations such as drug resistance;
- Overcoming biological barriers;
- Improving the sensitivity of diagnosis and imaging of tumorous sites;
- Linking anti-cancer active components with imaging molecules in order to attain a real-time assessment of the in vivo efficiency of the drugs;
- Developing new paths for the manufacture of synthetic vaccines; and
- Improving cancer diagnosis and imaging with scaled-down medical devices.

All the advancements in nanomedicine detailed above shed some light on another very important aspect: the complexity of the cancerous sites and the challenges that need to be overcome, as well as all the possibilities that come from that discovery. Generally, nanoparticles loaded with therapeutic agents are delivered systematically, in the case of solid cancer therapy. These nanoparticles agglomerate in the cancerous site as a consequence of the enhanced permeability and retention effect (EPR), which has been known to be a result of poor vasculature in the tumor site. However, although it sounds somewhat simplistic at first, EPR is a very complex process that can be influenced by several biological actions made by the systematic distribution of nanoparticles. Interactions between proteins and nanoparticles, blood flow, and the tumor microenvironment are all factors that can influence EPR. The EPR effect can also be manipulated by the nanoparticles’ features, such as their dimensions, shape, surface properties, porosity, and structure [6].

It is very important to understand that most of the present insight on the in vivo behavior of nanoparticles is built on data attained from animal studies. Even though there have been clinical and preclinical studies on the action mechanism of nanoparticles containing pharmacological active compounds, it is difficult to correlate the data in between species [6].

## 2. Nanomaterials for Drug Delivery

The advancements made in fields such as nanomedicine and nanotechnology have led to significant progress in the development of nanomaterials that can be used in medical-related applications such as therapeutic, diagnosis, and imaging [7]. Over the past years, drug delivery systems using nano-scaled polymeric or inorganic nanoparticles have been intensively researched because of their potential in oncological applications. By comparing with classical anti-cancer treatments, nanomaterials bring unique properties that can overcome the disadvantages of conventional therapies, such as lack of specificity and high drug concentrations [2].

Nanomaterials are considerably smaller in size than biological macromolecules. More exactly, they usually have a diameter of tens of nanometers (nm), which makes them from 100 up to 1000 times smaller than even one cancer cell. Therefore, as compared to microparticles, nanomaterials display superior intracellular uptake, which makes them suitable as vehicles for cancer drug delivery [8,9]. Moreover, due to their small sizes, nanomaterials have the potential to overcome the biological barriers. For example, the smaller capillaries have sizes around 3 micrometers in diameter. Nanomaterials that are specifically engineered to have a maximum size of 200 nm could take advantage of the circulatory system and get transported to a specific site and deliver pharmaceutically active agents [2].

Another advantage of their small size is their very large surface area. Due to their increased surface area, a larger quantity of drug molecules can be attached and delivered. As an example, a single polymeric nanoparticle that has an average diameter of 70 nm can accommodate roughly 2000 drug molecules, as compared to polymer–drug conjugates where only nine drug molecules can be contained in each molecule. The increased drug loading volume comes as an excellent advantage for cancer drug delivery therapies. Moreover, binding specific targeting ligands increases the chances for effective drug delivery [2].

Most importantly, nanomaterials could overcome the limitations of conventional cancer treatments. The main disadvantage of chemotherapeutic drugs is their insolubility in water, for example paclitaxel. Paclitaxel is one of the key chemotherapeutic drugs used for treating several types of cancer due to its ability to induce cancer cell death. However, its main drawback comes from the above-mentioned poor solubility, which has proven to be a challenge when developing stable formulations for cancer treatment. In order to overcome this limitation, anti-cancer drugs can be incorporated into polymeric nanoparticles that can improve water solubility. Moreover, the encapsulating process also improves the bioavailability and efficiency of the therapeutic activity. As an example, conventional drugs that are characterized by a low molecular weight are very quickly cleared by the renal system, and therefore, insufficient drug concentrations reach the desired site. In comparison, incorporating these drugs in nanoparticles significantly increases their blood circulation half-life, resulting in the efficient delivery of the drugs to the targeted area. Another advantage of using nanoparticles drug delivery instead of conventional chemotherapeutic delivery is that when encapsulated in the nanoparticles, the drugs are protected against metabolic degradation, and their toxicity toward the surrounding tissues is controlled. Recent research has been focused on stimuli-responsive nanomaterials that have the potential to further improve the efficiency of targeted drug delivery in cancerous sites [2].

### 2.1. Properties of Nanoparticles

There are some characteristics that nanoparticles need to posses in order to achieve an effective system for cancer therapy. First of all, the nanoparticles must be biocompatible, of high bioavailability, and stable at physiological conditions. They also need to be compatible with the pharmaceutically active drug or drugs used in order to bind and transport them. Moreover, the nanoparticles need to be capable of targeting the specific site, without damaging surrounding healthy tissues or cells. Lastly, they need to be able to release the load once they reach the targeted site. These characteristics can be largely affected by the physicochemical properties of the nanoparticles [10].

Size, for example, has a major effect on the ability to penetrate tumors. Due to their leaky vasculature, the nanoparticles’ size can be engineered to be small enough to enter the tumor, but at the same time big enough to prevent extravasation from normal blood vessels (one of the strategies exemplified in Figure 2). This aspect is very important, because it prevents agglomeration in unwanted parts of the body [10]. However, different organs have different size uptake specifications, making it even more challenging for the design of nanoparticles. Many studies have been made to determine the proper size of nanoparticles for tumor accumulations, and it was observed that it is the main factor that determines the penetration inside a tumor site. Investigations on silica nanoparticles showed that smaller-sized particles (50 nm) exhibited high anti-tumoral efficiency than larger-sized particles (200 nm) [11]. Moreover, in comparison to micron-sized particles, nanoparticles displayed improved bioavailability and degradation rates [12].

Shape is another important factor when designing nanoparticles for cancer therapy, because it influences fluid dynamics. It has been observed that the shape of the nano-sized carrier can govern the interaction between the membranes of the cells and the nanoparticles. Regarding the biological barriers, it was noted that shape is one of the major factors that influences whether the nanoparticles are taken up by the reticuloendothelial system (RES). As a result, this affects the blood half-life, and can either increase or decrease the chances of the system to reach its desired site. The majority of the nano-sized carriers for cancer applications that have been investigated are designed in a spherical shape, most probably because of the challenges in synthesis [13,14,15].

Surface chemistry can include surface charge, porosity, and alterations, which all have an impact on the fate of the nanoparticle. Many of the system properties, such as surface interactions, degradation or agglomerations rates, and cellular uptake are influenced by surface chemistry. Most studies suggest that having a positively charged surface increases the chances of cellular uptake. The surface can also be altered in order to increase circulation through blood [12,14]. However, different surface functionalities can be required for different types of cancers or even different stages of the same cancer type. For example, Srivastava et al. studied the effect of neutral, anionic, and cationic charges for carbon nanoparticles on breast cancer cells. The results indicated that for metastatic phases, the anionic phosphate nanoparticles exhibited better targeting activity, whereas for late phases, sulfonate-functionalized nanoparticles presented increased drug delivery efficiency [16].

### 2.2. Cancer Theranostics

Theranostics is a novel term that is used to describe systems that can provide both diagnosis and treatment. Actually, theranostic nanomedicine is basically the use of nanomedicine to prepare these kinds of systems. Therefore, this system can diagnose, provide targeted delivery, and monitor the effects of the treatment. It is expected that bringing together these aspects would lead to improvement over disease management, as well as minimize the risks and the expenses. All diseases could benefit from rapid diagnosis and treatment. However, due to the high morbidity rate of cancer, initial research of theranostics has focused on oncology. However, designing theranostic agents requires vast knowledge of several aspects, such as the properties of the materials used, their compatibility, and toxicity concerns. The purpose is to develop a nano-sized system with dual function. Therefore, it is of high interest to take into consideration all the steps required to develop such a system, starting from the methods and the materials used in the preparation of the initial particles to the biological barriers that they will encounter, and their elimination from the body [17,18,19].

In order to attain an effective theranostic system, various challenges have been acknowledged. Most importantly, the biomarker that is used for both imaging and treatment should have two main characteristics: (1) it should be overly expressed in the cancerous cells, and (2) it should be, preferably, absent in healthy cells. Moreover, the targeting ligand that connects with the receptor needs to be highly reproducible for in vivo testing purposes. It is also very important that the nanoparticles that are used in the development of the theranostic agents are biocompatible, biodegradable, and present a high loading capacity, as well as a controlled release profile of the therapeutic components when reaching the cancerous site. All the above-mentioned specifications are critical for the therapeutic part of a theranostic system; however, the diagnosis part mainly represents imaging requirements. The used nanoparticles should be able to produce a constant, clear imaging signal in order to effectively monitor the targeted drug delivery as well as the response [20,21].

## 3. Biological Barriers that Influence Drug Delivery

### 3.1. Tumor Microenvironment and Vasculature

The tumor microenvironment is known to act as a barrier to prevent drug delivery through several features, such as poor vasculature, high interstitial fluid pressure, and a dense extracellular matrix. Therefore, in order to attain efficient drug delivery for tumor combating, there is a need for the understanding the structure and specific aspects of its microenvironment [22].

**Figure 1:**
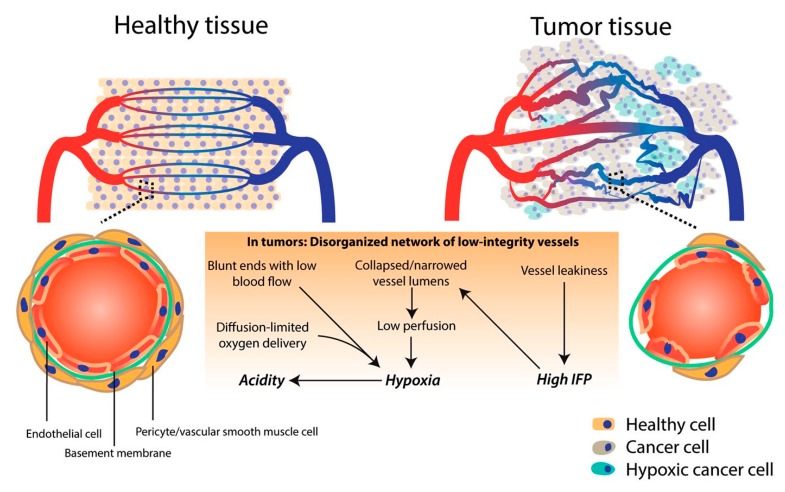
Schematic differences between healthy and tumor vasculature. Reprinted from an open access source (http://creativecommons.org/licenses/by/4.0/.) [23].

Angiogenesis is the development of new blood vessels that originate from already existing blood vessel structures. When discussing the vasculature of a tumor, it is known that it has an extra production of angiogenic factors, resulting in convoluted and leaky vessels (Figure 1). This particular aspect comes as an advantage for drug delivery, since the vasculature enhances the EPR effect, therefore allowing nanoparticles to be discharged from the vessels and accumulate inside the tumor. However, the same process that comes as an advantage for nanoparticle drug delivery can also be a disadvantage. Even though the structure of the vessels can extravasate the nanoparticles, it can also do the same for blood components, therefore blocking the extravasation of nanoparticles. Moreover, some areas inside the tumor suffer from a lack of perfusion, and therefore create an acidic and hypoxic environment, making the delivery of pharmaceutically active components even more challenging. Furthermore, the hypoxic environment leads to the advancement of the tumor, alongside with increasing its resistance, which translates into treatment failure [2,22].

The reason why the extracellular matrix of the tumor is so dense and the vasculature has this specific architecture is the high interstitial fluid pressure. Similar to the vasculature, high interstitial fluid pressure can both enhance the accumulation of nanoparticle-based drug delivery systems in the tumor, as well as obstruct their penetration. This inconsistency significantly hinders the favorable outcome of nanoparticles. In order to achieve penetration through the tumor, research has been focused on decreasing the interstitial fluid pressure so that drug delivery can be successfully attained [22].

The extracellular matrix is usually comprised of collagens, glycoproteins, proteoglycans, elastin, and hyaluronan, and its purpose is to act as a support for the development of cells and tissues. Due to the rich density of the extracellular matrix, the interstitial fluid pressure is increased and results in the limitation of fluid transfer, which decreases the chances of nanoparticles to penetrate through [22].

Another factor that challenges the delivery of drugs is solid stress. Solid stress is generated by the chaotic proliferation of cancer cells. Moreover, one of the main disadvantages of solid stress is that it weakens the immune response by augmenting tumor cell invasion [22].

### 3.2. Reticuloendothelial System (RES)

The reticuloendothelial system (RES) is one of the ‘components’ of the immune system. The RES is comprised of cellular and non-cellular elements, and is one of the biological barriers that challenge drug delivery. Cells and macromolecules can attach to the nanoparticles and generate an immune response that results in the hindering of the nanoparticles process when penetrating the targeted tissue. One of the means of avoiding RES is the surface functionalization of the nanoparticles, therefore increasing their circulation time in the blood circulatory system. Polyethylene glycol coated (PEGylated) nanoparticles have gained a lot of attention due to their ability to avert recognition by RES and increase their traveling time to the targeted site [14].

The dimensions and surface properties of the nanoparticles is a key factor in determining which part of RES will act upon them, such as the spleen, liver, and lungs. For example, hydrophobic surfaced nanoparticles are usually uptaken by the liver, the spleen, and then the lungs. On the other hand, nanoparticles that have a hydrophilic surface exhibit considerably less uptake by the liver and spleen. Currently, the research focus has shifted toward reducing the size of the nanoparticles, as it was proved to reduce the RES uptake [24]. Studies have been made on the effect of lipid nanoparticles as a drug delivery system. These nanoparticles have been PEGylated, and were tested on the delivery of siRNA in solid tumors. The tests were run onto xenograft models, and the results showed that these nanoparticles can possibly deceive the RES system and were capable of delivering up to almost 33% of the initial injected dose. Even though significant progress has been made in coating nanoparticles in order to partially trick the RES system, complete evasion has not been possible yet [25].

### 3.3. Blood–Brain Barrier (BBB)

The brain has a highly specialized protection structure called the blood–brain barrier (BBB), which is different from the structure of the other blood vessels. This barrier allows only specific biomolecules to pass through, and prevents the access of potential harmful agents. This whole process’s role is to protect the central nervous system from harmful agents and supply it with essential nutrients. However, when discussing the treatment for diseases of the central nervous system, such as cancer, the BBB can provide meaningful challenges [26].

The structure of a healthy BBB in mainly made up of brain capillary endothelial cells, and it is arranged under the shape of a ‘wall’ that goes around the brain capillaries. Specific transporters are the ones that deliver the essential biomolecules to the brain, whereas receptor-mediated trancytosis delivers larger molecules from the circulatory system to the brain [26].

Similar to the BBB formed by the healthy brain’s circulatory system, the convoluted vasculature of brain tumors generates a blood–tumor barrier (BTB). The brain tumor vasculature is hetrogenenous, and is mainly formed from capillaries that can have three different features: (i) they can be continuous and without perforations (similar to the vasculature of a healthy brain), (ii) they can be continuous and have perforations (increases the changed of permeability), and (iii) they can be non-continuous (they have interendonthelial gaps) [26].

In regard to drug delivery treatment, the level of drugs delivered is significantly increased in sites where perforations and non-continuous endothelial is displayed. This feature is generally found in the near proximity of the tumor core; however, even so, because of the heterogeneity of BTB, drug delivery and permeability is still challenging due to the invasive cells found at the margins of the tumor that are under the control of the BBB [26].

The inefficiency in the delivery of pharmaceutically active agents to the invasive cells following the BBB is one of the main reasons for tumor recurrence, despite initial surgical interventions for tumor removal [26].

The current methods that are used in order to attain increased permeability include direct injections, either intraventricular or intracerebral, infusion, and even implantation. However, these methods are associated with risks such as high toxicity and inadequate drug distribution [14].

Therefore, research has been focusing on alternative ways to attain the desired results without the disadvantages of the above-mentioned methods. One approach for nanoparticles to go past the BBB is receptor-mediated endocytosis. More exactly, this approach is based on conjugating receptors with ligants or peptides that then attach the nanoparticles to the exterior area of endothelial cells. Using nanoparticles for the delivery of drugs to the brain tumor presents several advantages, including increased drug circulation [27]. This can enhance the interaction between the drugs and specific molecules, and therefore pass over the BBB. Moreover, nanoparticles can be designed to present specific functionalities. Their surface architecture, as well as their size, can be tailored in order to enhance their capacity to overcome the BBB. Gold nanoparticles have gained attention due to their advantages such as chemical stability, optical features, and diverse size synthetization. Most importantly, their surface can be easily functionalized with several peptides, proteins, and other biomolecules. Moreover, in vivo computed tomography (CT) imaging can be used to visualize gold nanoparticles in a non-invasive manner [28].

### 3.4. Kidney Filtration

The role of the renal system is to filter the circulating blood, which is an important aspect when engineering nanoparticles for drug delivery. Several properties of the nanoparticles need to be taken into consideration, such as the size, shape, and surface charge, because they can all affect the clearance ratio in the kidneys. For example, it has been observed that even a small change in size of only 2 nm (from 6 to 8 nm) can make a difference regarding kidney clearance—the smaller the size, the higher the renal clearance [29,30]. For example, despite the large molecular mass of single-walled carbon nanotubes (SWCNs), specifically designed rod-like SWCNs of around 1 nm in diameter were shown to infiltrate through the perforated capillary endothelium [14,31,32].

However, enhancing renal clearance and attaining delivery efficiency are two different aspects that are not co-dependent. Several approaches have been investigated, including the synthesis of biodegradable nanoparticles that can break down into biocompatible by-products that can easily go through renal clearance. This method can also come with disadvantages, such as non-specific degradation, as well as the delivery of drugs before reaching the desired tumor site. The levels of the pharmaceutically active component must be maintained when traveling through the plasma in order to attain the desired chemotherapeutic effect. Moreover, bloodstream clearance must be safely achieved so that the possibility of long-term adverse effects is confined. This specification becomes even a more challenging aspect for patients that are already suffering from kidney deficiencies, for whom the need for personalized treatment arises [14,31,33].

**Figure 2:**
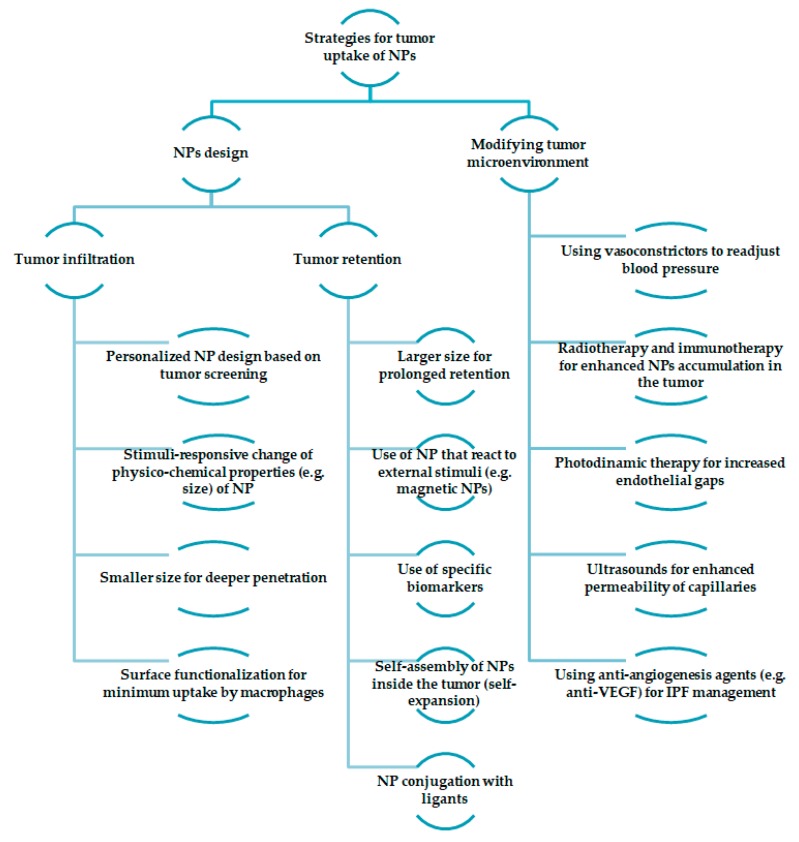
Different strategies for tumor uptake of nanoparticles.

Figure 2 highlights some of the strategies for the tumor uptake of nanoparticles. There are two different approaches: nanoparticles design and modification of the tumor microenvironment. Furthermore, the design of the nanoparticles can be either obtained in regard to tumor infiltration or retention. For tumor infiltration, several strategies have been noted: personalized nanoparticle design based on tumor screening [34], stimuli-responsive change of physicochemical properties of nanoparticles [35], and smaller sizes that result in deeper penetration and surface functionalization for minimum uptake by macrophages (for example, PEGylation) [36]. On the other hand, when discussing tumor retention, the size of the nanoparticles should be large enough for prolonged retention. Moreover, stimuli-responsive nanoparticles can be used for the same purpose, as well as the use of biomarkers, or conjugation with ligants [37]. Another strategy would be the use of self-assembly nanoparticles inside the tumor [38]. Modifying the tumor microenvironment can be obtained through different approaches: the use of anti-angiogenesis agents in order to manage the interstitial fluid pressure (IPF) [39], vasoconstrictors for blood pressure readjustment [40], ultrasounds for the enhanced permeability of capillaries [41], photodynamic therapy for increased endothelial gaps [42], and radiotherapy and immunotherapy for enhanced nanoparticle accumulation in the tumor [43].

## 4. Types of Nanoparticles

Over the last years, there have been tremendous accomplishments in the field of nanomedicine in respect to drug delivery. Especially for oncology-related applications, numerous types of nanoparticles have been developed in order to attain an efficient drug delivery system that also has specific functions for diagnostic purposes, apart from its therapeutic effects. Due to their particular properties, both organic and inorganic nanoparticles have been researched for this purpose [44]. Amongst all the types of nanoparticles, two examples from each, organic (liposomes and polymeric nanoparticles) and inorganic (gold and magnetic nanoparticles), have been detailed in this review due to their unique properties.

### 4.1. Liposomes

Since 1963, when they were first discovered, liposomes have been intensely studied due to their specific properties and the advantages that they can employ as drug delivery vehicles. They can be synthesized from cholesterol and phospholipids, and are well known for their self-assembling properties. One particular property has made them extremely valuable for drug delivery purposes, which is their amphiphatic nature; this characteristic enables them to bind both hydrophilic and hydrophobic compounds. More specifically, they can encapsulate water-soluble pharmaceutically active compounds in their core, and at the same time, nonpolar drugs in their bilayer membrane [45,46]. Apart from these, liposomes have several other beneficial properties for drug delivery, such as biocompatibility and biodegradability; they do not present toxicity or immunogenicity [44,47]. Most importantly, drug delivery systems based on liposomes have already been approved by the FDA for cancer therapy, such as for example Myocet^TM^ [48].

The first ever FDA-approved liposome-based drug system was Doxil^®^, in 1995. Since the nano-sized liposomes were PEGylated, the system exhibited a prolonged circulation time of the pharmaceutically active component, as well as RES evasion. The system also showed a high loading capacity of Doxorubicin and targeted drug delivery at the tumor site [49].

Zhang et al. developed a formulation based on liposomes and paclitaxel for cancer drug delivery applications. The lyophilized system exhibited encapsulation efficiency over 90%, whereas the dimensions of the particles were around 150 nm, both before and after lyophilization. Moreover, the system showed physical and chemical stability for 12 months. When diluted, the size of the particles did not show change, and the drug remained encapsulated. Furthermore, in physiologic conditions, the system exhibited stability, and did not release significant quantities of the drug within 120 h [50].

Zhao et al. studied the anti-tumoral effect of a pH-responsive liposome containing system on glioma tumor cells. Due to the acidic pH of gliomas, the system was composed of a tumor-specific pH-sensitive peptine and liposomes, which could respond to the acidic environment and release the encapsulated drug. Doxorubicin was used as an anti-cancer drug. The results from both in vivo and in vitro studies showed that when the system reached acidic pH, it released the pharmacologically active component. Therefore the system exhibited stimuli responsive behavior to acidic pH, proving to be a suitable candidate for anti-cancer treatments [51].

Theranostic systems based on liposomes that can be used for both imaging and drug delivery purposes have been investigated. For examples, Ren et al. designed such a system, encapsulating a pharmaceutically active component, whose biodistribution could be imaged in real time by magnetic resonance imaging (MRI) and achieve chemotherapy. When compared to the commercially available MRI contrast agent Omniscan^®^, the designed system exhibited better results, as well as a longer circulation time in vivo. Moreover, the liposome allows both polar and nonpolar chemotherapeutic drugs entrapment, therefore providing synergetic therapy with sustained release and considerably lower toxicity [52].

### 4.2. Polymeric Nanoparticles

It has been considered that polymeric nanoparticles may be very efficient vehicles for prolonged drug delivery systems. The use of polymeric nanoparticles for controlled release related applications has been studied since the early 1970s. Two decades later, polymeric nanoparticles synthesized using PLA (poly(lactic acid) and PLGA (lactic-*co*-glycolic acid) were investigated and described as ‘long-circulating’ [53]. This discovery has resulted in increasing interest toward polymeric nanoparticles and their use in therapy-related applications. The main advantage of using polymeric nanoparticles is their versatility; they can be engineered to be either biodegradable or non-biodegradable, and moreover, they can be either synthetic or derived from natural sources [54,55].

One of the benefits of biodegradable polymers is that they break down into separate monomers, which can be easily eliminated from the body by its natural metabolic pathways. The physicochemical features of the polymer (e.g., density, molecular weight, crystallinity, and hydrophobicity) are determining factors for the degradation rate and the kinetics of the release of the drug [54].

Both synthetic and natural polymers have been investigated for targeted drug delivery applications over the last years. Natural polymers such as polyhydroxyalkanoates (PHAs), as well as synthetic, poly-lactide-co-glycolide (PLGA) have been investigated in this regard. Coupled with anti-cancer agents such as paclitaxel [56,57], doxorubicin [58,59], cisplatin [60,61], and many others, these systems have been tested in vivo, and some have even been used in preclinical trials on mice [62].

The use of folic acid was investigated for the treatment of prostate cancer using PLGA nanoparticles functionalized with chitosan as vehicles. The folic acid-conjugated nanoparticles were then loaded with bicalutamide and tested in vitro. In order to highlight the advantages of the system, unfunctionalized PLGA nanoparticles were synthetized and subjected to the same tests. Cytotoxicity tests showed that the functionalized nanoparticles exhibited improved efficiency compared to the unfunctionalized ones due to their altered surface and specific targeted delivery [63].

Poly(3-hydroxybutyrate-co-3-hydroxyoctanoate) nanoparticles conjugated with folic acid and loaded with doxorubicin displayed a drug encapsulation performance of above 80%. Moreover, in vitro assays showed a release profile of the anti-cancer agent of almost 50% in the first five days. In vivo testing revealed that the system exhibited enhanced therapeutic efficiency in restraining the growth of the tumor, when compared to controls [58].

PLGA nanoparticles loaded with methotrexate–transferrin conjugates were studied as vehicles for brain cancer therapy. The above-mentioned nanoparticles were further coated with a water-soluble surfactant called Polysorbate 80, which is known to enhance the BBB cross of nanoparticles loaded with active molecules. The sustained delivery of methotrexate–transferrin conjugates was attained due to the over-expressed transferrin receptors on the surface of cancerous cells. Experimental results for both in vivo and in vitro assays highlighted the anti-tumor capability of the novel system compared with controls [64].

### 4.3. Gold Nanoparticles

Gold nanoparticles have received a great deal of attention for cancer-related applications due to their ability to thermally affect and destroy cancerous cells, as well as their photothermal heating capacity and ease in surface functionalization. One of the classical approaches for cancer therapy, namely hyperthermia, relies on heating up to 40 °C the tumor containing location of the body. Ultrasounds, microwaves, and radio waves are the main external heat generators. However, using gold nanoparticles as the heat source comes with several advantages over traditional hyperthermia. The main advantage is that when using gold nanoparticles, the heating only affects the site adjacent to the nanoparticles, without harming other healthy tissues or cells. Moreover, the temperature used can be significantly higher over short periods of time. All these result in a targeted action specifically on the site of interest, therefore reducing some of the main disadvantages of classical approaches [65,66].

When an external radiofrequency electric field is acted upon the internal gold nanoparticles, it was observed that they start heating up the adjacent areas. Therefore, they are potential candidates for radiofrequency hyperthermia. It has been reported that subjecting cancer cells to radiofrequency hyperthermia can induce cell death in different types of cancers, but this method also has serious drawbacks, such as high levels of pain for the patients. Therefore, researchers have been investigating gold nanoparticles for their use in radiofrequency hyperthermia in in vitro cancer therapy of several cancerous cell lines [65].

Researchers have established the potential of gold nanoparticles for their use in radiosensitizers. It was noticed that gold nanoparticles can enhance their effects. However, even though the preclinical trials had promising results, gold nanoparticle-based radiosensitizers have not yet made their way toward clinical studies [67].

Glioblastoma is one of the most threatening types of brain and central nervous system cancers. Clinically, the main course of action is surgical resection, after which radiotherapy and chemotherapy are employed. However, as mentioned earlier, these classical cancer treatments come with severe side effects. Due to properties such as strong absorption, gold nanoparticles were observed to generate local dose augmentation at the cancerous site. Moreover, engineering a system that contains both gold nanoparticles and organic molecules, such as bovine serum albumin (BSA), results in a higher agglomeration of nanoparticles in the tumor. In addition, BSA-modified gold nanoparticles exhibit better features, including uniform dimensions, ease in synthesis, and stability under physiological conditions. Chen et al. evaluated the capacity of BSA-modified gold nanoparticles as radiation sensitizers for glioblastoma treatment. Both in vitro and in vivo assays showed promising results. The system exhibited inhibition of cloning formation, tumor cell death, and most importantly, did not present destructive consequences on adjacent tissues [68].

The principle of using nanoparticles for cancer therapy can be, in a general manner, quite simple: the nanoparticles—in this case, gold nanoparticles—are injected in the cancerous area, and when the external X-ray source acts upon them, they produce radicals that harm the tumor cells and promote cell death. In regard to radiotherapy, studies were made on intravenously injected gold nanoparticles in mice with the EMT-6 cancerous cell line. Then, the mice were subjected to X-ray therapy, and it was observed that the survival rate was substantially increased when compared to mice that were only treated with irradiation. Even though significant progress was achieved using unfunctionalized gold nanoparticles, even more advantages can be attained when linking these nanoparticles to specific biologically active reagents. Therefore, the molecule functionalization of nanoparticles is widely used in order to enhance the properties of the system. In this regard, Kong et al. synthesized gold nanoparticles coated with cysteamine and thioglucose, and tested them on healthy and malignant breast cell lines. It was observed that the glucose-coated nanoparticles were internalized by the tumor cells, whereas the cysteamine-coated ones were mainly attached to the surface. However, the number of internalized functionalized nanoparticles was significantly higher than the unfunctionalized gold nanoparticles. The initial testing showed that these nanoparticles do not affect the cells in a drastic manner. However, when irradiation was applied, it was observed that the cytotoxic effect was significantly higher than with unfunctionalized nanoparticles [69].

Another treatment that can exploit the advantages of nanoparticles is photothermal therapy. In this therapy, the nanoparticles that are inside the tumorous site are subjected to laser light and generate heat. The main type of nanoparticles associated with photothermal therapy is gold nanoparticles due to their unique advantages, amongst which the following are particularly important in this instance: tumor penetration due to their small diameters, the ability to convert light to heat, and the capacity to be specifically engineered to absorb near-infrared light, which is more effective for tissue penetration than other wavelengths of light. Combining photothermal therapy with other cancer treatments, such as chemotherapy, in order to enhance the anti-cancer effects, has been intensely studied [70].

### 4.4. Magnetic Nanoparticles

Nanotechnology and the recent advancements in this field have provided development opportunities for novel imaging and therapeutic agents with exceptional properties that can reduce the adverse side effects of conventional treatments for cancer and provide specificity. Even though several types of nanomaterials have been investigated, magnetic nanoparticles have gained significant attention for numerous applications in the biomedical field varying from imaging to targeted drug delivery. Most importantly, magnetic nanoparticles originating from iron oxide have been FDA-approved in magnetic resonance-based applications as contrast agents. However, there are several features of these nanoparticles—such as their size, size distribution, and shape—that have the potential to affect their magnetic properties. Furthermore, another important aspect for their use in biomedical applications is their functionality. This aspect is also affected by the above-mentioned parameters, as well as by their surface features. Apart from the unique properties that come along with the use of magnetic nanoparticles, especially in cancer therapy applications, they can gain additional features, such as biocompatibility and physiological stability when functionalized [71].

There has been a lot of struggle in research in the attempt to optimize cancer treatments. However, recently, focus has shifted toward the engineering of nanosystems with combined therapeutic properties, which is believed to be a promising approach. Traditional hyperthermia treatments implied high levels of heat since cancer cells are more easily harmed by heat than healthy cells. Implanted probes that can be remotely activated have been investigated, but experimental results from nanoparticles used as thermal agents indicated that their advantages might be superior. Colloidal suspensions can be attained using magnetic nanoparticles, making them easy to inject and therefore avoiding the surgical need for macroscopic implantation. Moreover, due to their small dimensions, they can overcome the biological barriers and generate the required thermal treatment in the proximity of the targeted site, without severe damage to healthy cells and tissues. Thermal agents are activated by light or magnetism, whereas photothermal agents are activated by near-infrared light, generating less damage to the tissue. However, both approaches suffer from their own disadvantages [72].

Fortunately, it has been investigated that iron oxide nanoparticles have the ability to act as both a magnetic and a photothermal agent. Espinosa et al. reported the analysis of iron oxide nanocubes as dual agents. It was observed in aqueous suspension that when these nanoparticles are exposed to both a magnetic field and a near-infrared laser irradiation, it enhanced their heating ability compared to only magnetic stimulation. When tested on tumor cells, the results indicated complete apoptosis of the cells. As a comparison, in in vivo testing, when only one of the treatments was employed in solid tumors, the result was the inhibition of tumor growth; however, when the dual mode was activated, the results showed complete tumor regression [72].

In order to achieve a significant therapeutic effect on a specific ailing tissue without harming the surrounding tissue or cells, efficient and targeted drug delivery to the site must be attained, which has proven to be challenging for traditional cancer therapies alone. Nanomedicine comes with several advantages over traditional approaches regarding drug delivery, such as targeting the tumorous site either by active or passive approaches, overcoming biological barriers, which increases the circulation of the drug toward the targeted site, sheltering the therapeutic agent from degradation during the delivery, controlled delivery, and engineering nanosystems that can deliver multiple drugs in cases of drug resistance. For effective drug delivery, magnetic nanoparticles are required to present adequate loading capacity, efficiently conserve the bioactivity of the drug, and deliver the therapeutic agents at the desired site without damaging the surrounding healthy tissues or cells [73,74].

Frounchi et al. synthesized a system composed of magnetic nanoparticles, namely magnetite (Fe_3_O_4_) coated with poly(lactic acid)/poly(ethylene glycol) (PLA/PEG) and loaded with doxorubicin. The system proved controlled drug release through both drug diffusion and PLA hydrolysis. Moreover, it was observed that PEG enhanced the drug release when the external magnetic field was activated [75].

Bhattacharya et al. investigated the use of magnetic nanoparticles in hybrid systems for dual-responsive drug delivery, thermal and pH sensitivity. In order to achieve a dual-resposinve system, the nano-sized system was comprised of magnetic nanoparticles and a polyethyleneimine cross-linked Pluronic F127 co-polymer. Moreover, to address cancer imaging and targeting, folic acid, rhodamine isothiocyanate, and doxorubicin were bound to the nanoparticles. This complex nano-sized system proved to be stable at physiological conditions, whereas at acidic pH, the results indicated an improved delivery of Doxorubicin (DOX). Fluorescence microscopy alongside with MRI was used to show their potential as imaging agents due to the folic acid receptor. Cytotoxicity analysis on an immortal human cell line (HeLa) cells showed that the system exhibited efficient therapeutic effects [76].

Parsian et al. studied the delivery of gemcitabine, a chemotherapy drug, when loaded onto iron oxide nanoparticles coated with chitosan. Drug-release assays showed that gemcitabine has a release rate at an acidic pH that is eight times higher than at neutral pH, which is desired in the delivery of pharmaceutical agents in a cancerous site. Moreover, comparing loaded gemcitabine onto nanoparticles with free gemcitabine on MCF-7 breast cancer cell lines, the results indicated the higher potential of the system [77]. Table 1 highlights the recent studies attained of the above-mentioned organic and inorganic nanoparticles in cancer therapy.

## 5. Conclusions

This review sums up the important parameters that need to be taken into account when designing systems for cancer therapy while considering the desired characteristics of the nanoparticles, as well as a biological point of view. The need for a system that can simultaneously diagnose, provide targeted delivery, and also monitor the effects of the treatment has shed light on a novel approach called theranostics. Even though there are challenges when developing such a system, research is ongoing, especially in cancer therapy, due to its high mortality rate.

When discussing the biological barriers that hinder drug delivery, it is important to mention the specific microenvironment and the vasculature of the tumor. It has been observed that some factors, such as the EPR effect, can act both as an advantage and a disadvantage for nanoparticle-based drug delivery. However, there are several other aspects that cause drawbacks such as poor vasculature, high intersititial fluid pressure, and dense extracellular matrix. Interestingly, similar to the blood–brain barrier, the abnormal vasculature of brain tumors develops a barrier of its own, called the blood–tumor barrier. Several approaches have been investigated in order to overcome these biological barriers.

Liposomes, polymeric, gold, and magnetic nanoparticles have been tested as potential candidates for cancer treatments. These nanoparticles exhibit impressive properties such as versatility, functionality, biocompatibility, and other specific features. Great improvements have been made so far for the use of nanobiomaterials in cancer therapy. However, there are still many challenges ahead, and an advanced understanding of the biological features is needed in order to design systems with tailor-made properties.

## Figures and Tables

**Table 1:** Recent studies on nanoparticles for cancer therapy.

Reference	Year	Type of Nanoparticles (NP)	Use	Results
**Lyposomes**				
Muhammad et al. [78]	2017	MRX_34_ – a liposoman formulation that mimics the tumor suppressor microRNA-_34_a	Phase I study for advanced solid tumors	Anti-tumoral activity
Bharti et al. [79]	2017	Liposome encapsulating diacerein (therapeutic molecule) and decorated with synthetic somatostatin analogue (receptor overexpressed in breast cancer cells)	Breast cancer therapy	Enhanced circulation time and apoptosis in breast cells, tumor growth inhibition
Kang et al. [80]	2017	Liposomal system loaded with dihydroartemisinin and doxorubicin	Co-delivery system for drug-resistant colon cancer therapy	High cytotoxicity to cancer cells and tumor inhibition rate above 80% compared to free drugs
Zhang et al. [81]	2017	Cisplatin-prodrug-constructed liposomes loaded with an antioxidant enzyme (catalase)	Enhanced chemoradiotherapy	Tumor hypoxia relief and DNA damage in cancer cells
Wang et al. [82]	2017	Chitosan-modified liposomes loaded with resveratrol and coated with gold nanshells	Chemophotothermal cancer therapy	High stability and photothermal conversion capacity, higher therapeutic effect on cancer cells compared to single therapies
**Polymeric Nanoparticles**				
Chen et al. [83]	2017	NIR800 polymer	In vivo imaging	High-contrast imaging of lymph nodes and tumors
Cui et al. [84]	2017	Polyethylene glycol (PEG)-grafted poly(cyclopentadithiophene-alt-diketopyrrolopyrrole) semiconducting polymeric NPs	Photoacoustic imaging agents	Imaging of tumor in living mice with a high ratio of tumor signal to background
Zhang et al. [85]	2017	Biocompatible electron donor–acceptor conjugated semiconducting polymer nanoparticles (PPor-PEG) with light-harvesting unit	Photoacoustic imaging-guided photothermal therapy	Complete tumor regression in tumor-bearing mice
Cheng et al. [86]	2017	Polydopamine-modified mesoporous silica NPs coated with poly(ethylene glycol)-folic acid, loaded with doxorubicin	Drug delivery system for cervical cancer therapy	High targeting efficiency and anti-tumor efficacy in vivo
Xu et al. [87]	2017	NP platform containing mitoxantrone core, poly(ethylene glycol) shell, and arginylglycylaspartic acid (RGD) peptide	Reactive oxygen species (ROS)-responsive polydrug NPs	Responsive to intracellular ROS and significant inhibitory activity on tumor cell growth
**Gold Nanoparticles**				
Cheng et al. [88]	2017	Diazirine-decorated gold nanoparticles	Photothermal therapy and photoacousting imaging of tumors	Negligible cytotoxicity, impressive photothermal ablation effect
Poudel et al. [89]	2017	PEGylated thermosensitive lipid-coated plasmonic hollow gold nanoshells, loaded with gemcitabine and bortezomib	Chemotherapy combined with photothermal therapy of pancreatic cancer	Efficient cellular uptake and apoptosis of cancer cells, specific drug delivery, exhibited photothermal effect
Wang et al. [90]	2017	Hollow gold nanoshell functionalized with small interfering RNAs against heat shock protein 70 (Hsp70)	Photothermal platform for induced hyperthermia therapy	Enhanced cellular uptake, efficient siRNA delivery and Hsp70 silencing
Yin et al. [91]	2017	Sialic acid-imprinted gold nanorods	Targeted near-infrared (NIR) cancer photothermal therapy	Biocompatibility, selectivity of targeted cancer cells and high photothermal effect
Zhang et al. [92]	2017	Anti-epidermal growth factor receptor-conjugated gold nanorods	Epidermal growth factor receptor therapy for triple-negative breast cancer using photoacoustic imaging-guided NIR photothermal therapy	Strong anti-proliferation, apoptotic activity of cancer cells, and tumor regression
Jia et al. [93]	2019	Gold-levonorgestrel nanoclusters	Radiosensitizer for enhanced cancer therapy	ROS production that leads to cell death significantly inhibited tumorigenicity after one treatment
Bera et al. [94]	2018	Porphyrin-coated gold NPs loaded with Doxorucibin (DOX)	Nanochemotherapeutic system	High encapsulation efficicency, selective internalization inside cancerous cells with increased retention time, targeted delivery,
Penninckx et al. [95]	2019	Amino-PEG functionalized gold nanoparticles	Radiosensitizer and effect on residual thioredoxin reductase	Rediosensitization effect dependent on cell type, thioredoxin reductase activity inhibition
Movahedi et al. [96]	2018	Folic acid-conjugated gold nanorods	Multimodal cancer therapy	Improved photosensitivity and radiosensitivity of cancerous cells, induced cell death in nasopharyngeal carcinoma cells (KB)
Mendes et al. [97]	2017	14-nm gold NPs loaded with DOX	Photothermal agents	Induced cell death in breast cancer cells
**Magnetic Nanoparticles**				
Rao et al. [98]	2017	Red blood cell membrane-derived vesicles-coated Fe_3_O_4_ NPs	Magnetic resonance imaging (MRI) and photothermal therapy	Enhanced stability and performance in in vivo MRI and photothermal therapy
Malekzadeh et al. [99]	2017	Fe_3_O_4_ NPs functionalized with poly citric acid, PEG, and folic acid	MRI for cancer therapy	Selective cellular uptake, increased NPs cytotoxicity on HeLa cells, enhanced magnetic resonance signal
Huang et al. [100]	2017	Superparamagnetic iron oxide NPs (SPIONs) coated with PEG, polyethyleimine (PEI) and folic acid and loaded with DOX	Drug delivery platforms for cancer theranostics	Low toxicity, specific targeting of cancer cell, and inhibition of tumor growth
Yang et al. [101]	2017	Hyaluronan-modified SPIONs	Breast cancer imaging and photothermal therapy	Specific cellular uptake and accumulation, meaningful contrast enhancement in MRI, concentration-dependent photothermal effect
Nosrati et al. [102]	2018	Fe_3_O_4_ conjugated with l-lysine and loaded with methotrexate	Drug delivery vehicle for breast cancer	Targeted delivery and internalization of the NPs, cytotoxic effect on human breast cancer cells, possible real-time montorization of drug delivery
Ghaznavi et al. [103]	2017	Au@Fe_3_O_4_ coated with PEG and folic acid	Photothermal therapeutic agent	Induced apopotosis in cancer cells
Nosrati et al. [104]	2018	Bovine serum albumin-coated Fe_3_O_4_ loaded with curcumin	Drug delivery carriers	Sustained release at body temperature, semnificative toxicity effect against breast cancer cells
Manatunga et al. [105]	2017	Fe_3_O_4_ coated with a bi-layer of sodium alginate and hydroxyapatite and loaded with curcumin and 6-gingerol	Delivery of hydrophobic drugs	High loading efficiency, sustained and controlled release at low pH
Trabulo et al. [106]	2017	Iron oxide MNPs conjugated with anti-CD47 antibody (CD47 – primary receptor expressed in pancreatic ductal adenocarcinoma) loaded with gemcitabine	Targeted delivery agent for pancreatic cancer cells	Selective and targeted delivery, induced apoptosis in pancreatic cancer cells
Mondal et al. [107]	2017	Fe_3_O_4_ coated hydroxyapatite NPs	Magnetic hyperthermia	No cytotoxicity without magnetic field, hyperthermia-mediated cell death on cancer cells
